# Supplementary material for: Differential B-Cell Receptor Signaling Requirement for Adhesion of Mantle Cell Lymphoma Cells to Stromal Cells
Source: Cancers (Basel). 2020 May 2;12(5):1143. doi: 10.3390/cancers12051143 (PMC7281289; doi:10.3390/cancers12051143)

Supplementray Materials: Differential B-Cell Receptor Signaling Requirement for Adhesion of Mantle Cell Lymphoma Cells to Stromal Cells

Laia Sadeghi, Gustav Arvidsson, Magali Merrien, Agata M.Wasik, André Görgens , C.I. Edvard Smith, Birgitta Sander and Anthony P. Wright


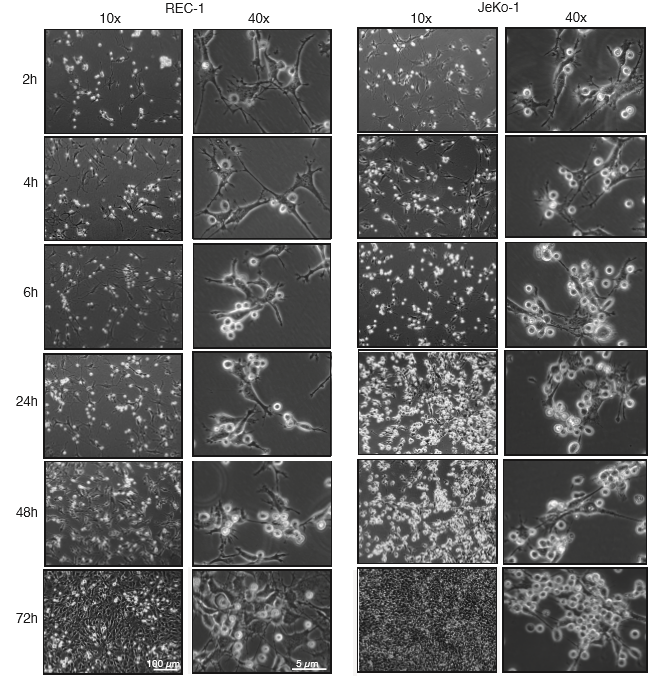


**Figure S1.** JeKo1 and REC-1 cells bind to stromal cells. Bright field microscopy images of co-cultured human JeKo-1 and REC-1 cells with MS-5 mouse stromal cells in indicate time points using 10x and 40x objectives.


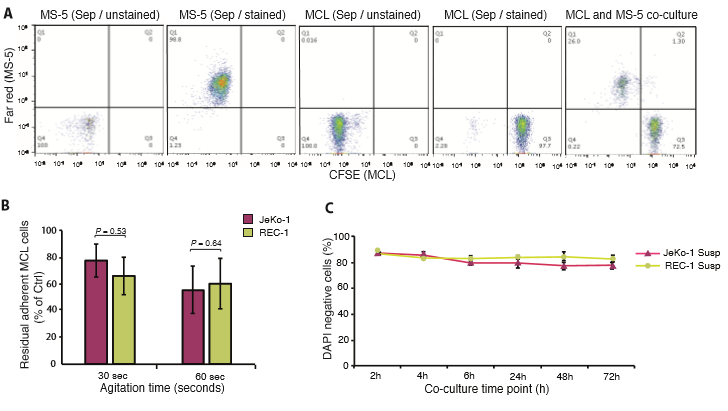


**Figure S2.** JeKo-1 cells bind more to stromal cells compared with REC-1 cells. (**A**) Flow cytometry gating strategies to separate MCL cells from MS-5 stromal cells in the co-culture. CFSE (Carboxyfluorescein succinimidyl ester) labeled MCL cells were co-cultured with Far Red label MS-5 stromal cells. Flow cytometry was used to separate two cell lines based on fluorescence signal. (**B**) MCL cells were co-cultured with MS-5 stromal cells for 4 hours. Suspension cells were removed and the number of adherent cells was quantified after washing for the indicated time periods (*n* = 3 representative of 3 independent experiments). Error bars show the s.e.m. Student’s t-test was performed and the *p* values indicate differences between JeKo-1 and REC-1 cells. (**C**) Suspension fraction of MCL cells in co-culture were stained with DAPI and the number of DAPI negative cells were calculated as percentage of total events.


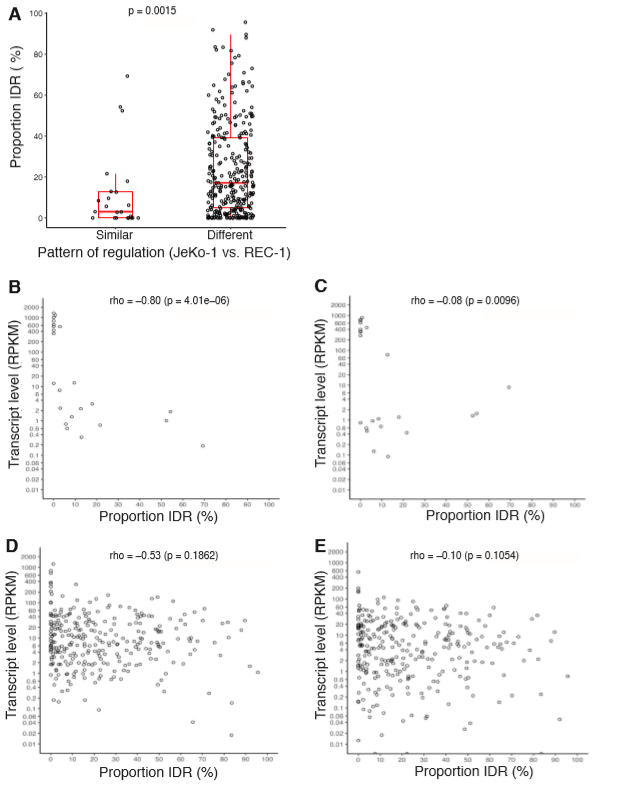


**Figure S3.** Intrinsic disorder region (IDR) content of proteins encoded by adhesion regulated genes in relation gene sets that are similarly or differently regulated in JeKo-1 and REC-1 cells. (**A**) Scatter plot comparing the IDR content (%) of proteins encoded by adhesion associated genes that are similarly or differently regulated in JeKo-1 and REC-1 cells upon adhesion to stromal cells. The box plot shows median and IQR values. Wilcoxon test *p*-value <0.05. (**B–E**) Scatter plots of estimated transcript levels (RPKM) in relation to the IDR content of encoded proteins for genes similarly (**B**, **C**) or differently (**D**, **E**) regulated genes upon adherence to stromal cells, using RPKM values for JeKo-1 (**B**, **D**) or REC-1 (**C**, **E**) cells. IDR scores were determined using the IUPred2A predictor (<https://iupred2a.elte.hu>) [[1](#_ENREF_1)] with the “long disordered region” argument. For genes associated with more than one protein sequence (e.g., splice variants) the first listed, longest retrieved sequence from Biomart was selected. Percent IDR content of proteins was defined as the percentage of residue-by-reside IDR scores >0.5. The RPKM values are the mean value (*n* = 4) for each cell line and condition, see Table S1.


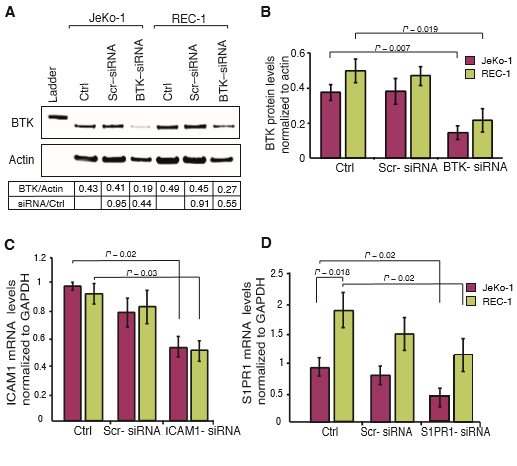


**Figure S4.** siRNA induced silencing of adhesion-associated genes. (**A**) The efficiency of BTK knockdown in JeKo-1 and REC-1 cells using siRNA was confirmed by western blot using actin as loading control. Total BTK was normalized to actin and BTK levels in transfected cells were normalized to non-transfected Ctrl. (**B**) Western blot data showing BTK protein levels in 3 independent experiments normalized to actin. (**C**) ICAM1 mRNA levels in non-transfected, scrambled and ICAM1-siRNA transfected cells normalized to GAPDH. (**D**) S1PR1 mRNA levels in non-transfected, scrambled and S1PR1-siRNA transfected cells normalized to GAPDH. Error bare represent the s.e.m. Student’s t-tests were performed and the *P* value indicate differences between un-transfected Ctrl and transfected cells.


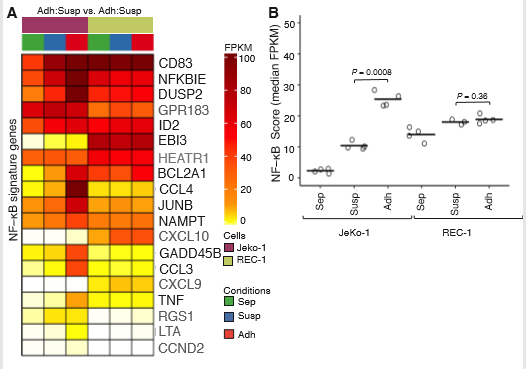


**Figure S5.** Differential expression differences in NF-κB signature genes in JeKo-1 and REC-1 cells upon adhesion to stroma. (**A**) Transcript levels for NF-κB signature genes for three different fractions of JeKo-1 and REC-1 cells, mono-cultured cells (Sep) in green, suspension cells in co-culture (Susp) in blue and adherent cells in co-culture (Adh) in red (*n* = 4 independent experiment). (**B**) Median FPKM values (Fragments Per Kilobase per Million of reads) for NF-κB signature genes for mono and co-cultured (Susp and Adh) JeKo-1 and REC-1 cells. (1.59 × 10^–11^) Fisher’s exact test *P*-value for the intersect between differentially expressed genes (FDR q-value ≤ 0.05 and absolute fold change ≥ 1.5) and NF-κB signature genes**.**


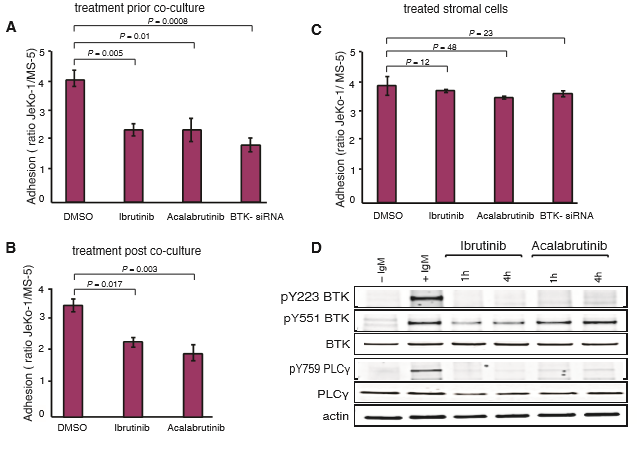


**Figure S6.** BCR signaling is important for adhesion of JeKo-1 but not REC-1 cells to stroma. (**A**) JeKo-1 cells were treated with 0.5 μM Ibrutinib or 1.5 μM Acalabrutinib for 1 hour, then treated cells as well as BTK knockdown JeKo-1 cells were co-cultured with MS-5 cells for 4 hours and the number of adherent cells were quantified using flow cytometry. (**B**) JeKo-1 cells were co-cultured with stromal cells for 24 hours, then un-bound cells were removed and 0.5 μM Ibrutinib or 1.5 μM Acalabrutinib or normal medium were added to the co-cultures for 4 hours and the number of adherent cells was quantified using flow cytometry. (**C**) MS-5 stromal cells were treated with 0.5 μM Ibrutinib or 1.5 μM Acalabrutinib for 1 hour, treated cells as well as BTK knockdown (BTK-siRNA) cells were subsequently co-cultured with JeKo-1 cells for 4 hours and the number of adherent cells was quantified using flow cytometry. (**D**) Western blot data showing total BTK, serine 223 and serine 551 phosphorylation levels in Ibrutinib and Acalabrutinib treated cells 1 and 4 hours after treatment. Error bare represent s.e.m. Student’s t-tests were performed and the *P* values indicate differences between Ctrl and BTK blocked cells.


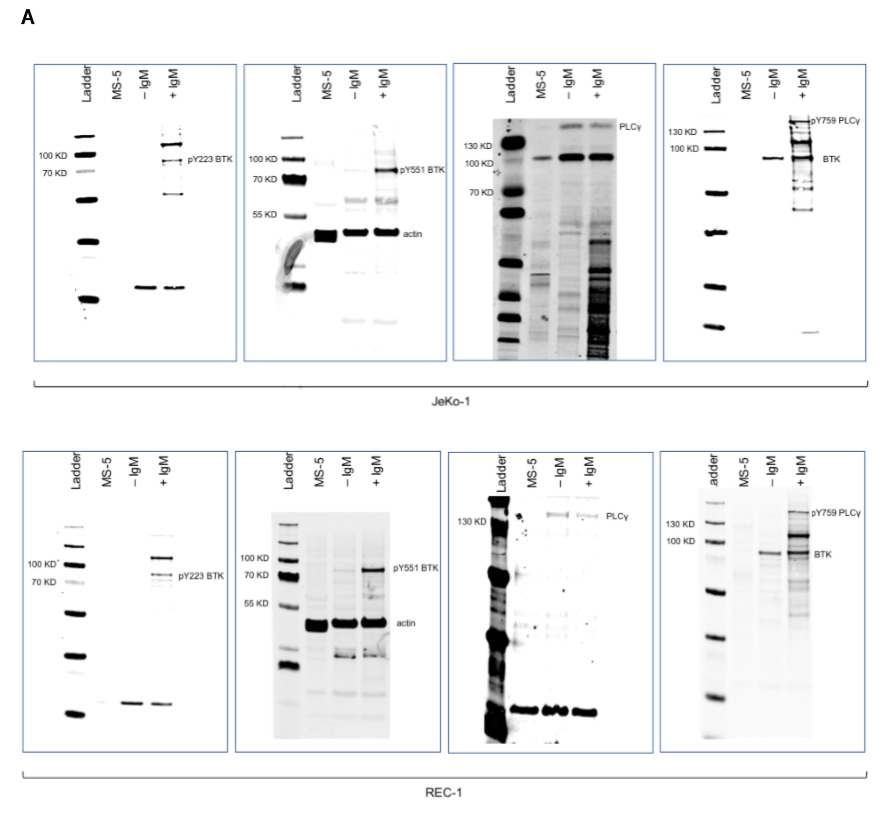


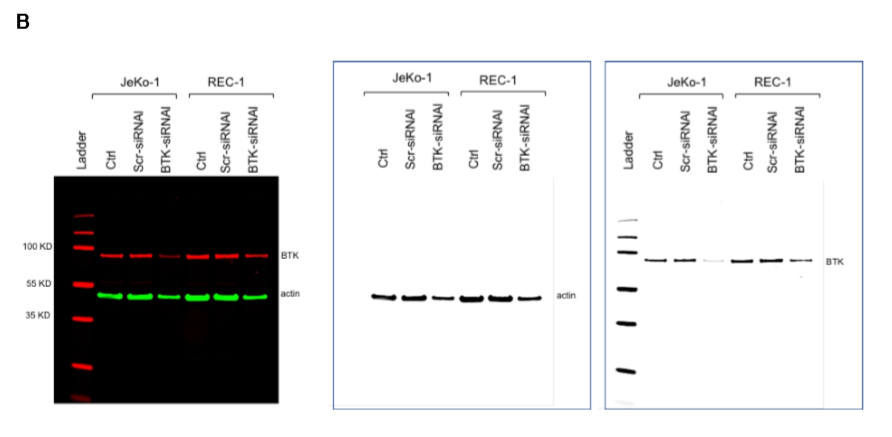


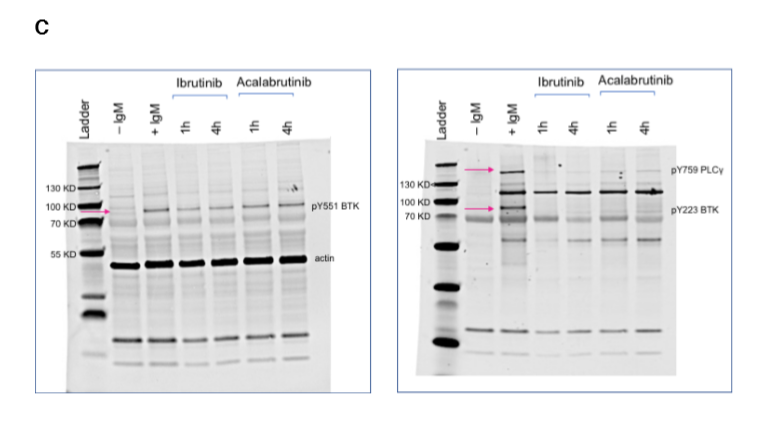


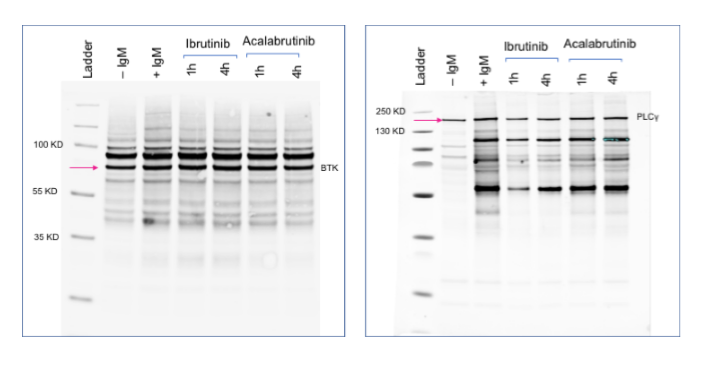


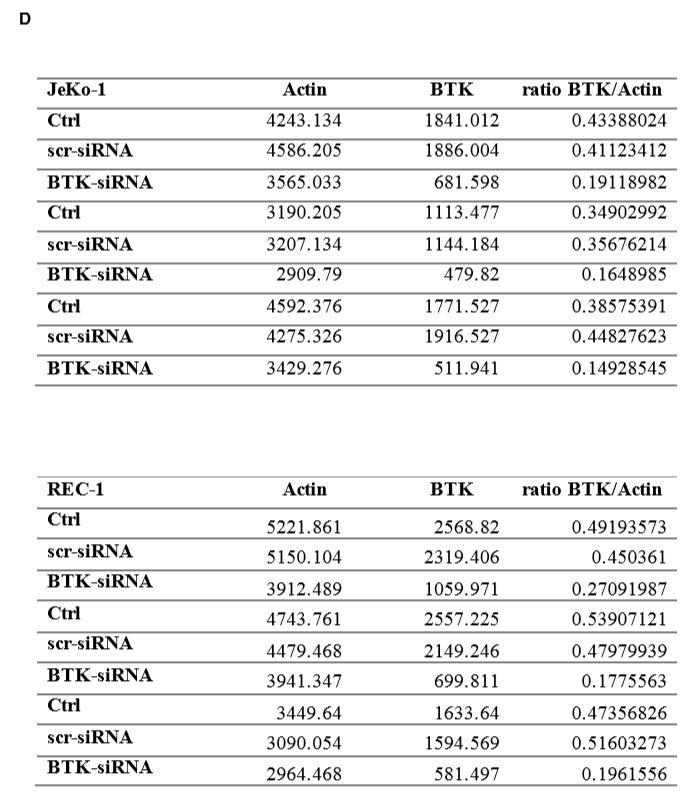

Supplement: Supplementary file 1 [file cancers-12-01143-s001.zip › cancers-771157-suppl/cancers-771157-Supplemetary materials Figure S1-S7.docx]
